# Contactless transient carrier spectroscopy and imaging technique using lock-in free carrier emission and absorption

**DOI:** 10.1038/s41598-019-49804-8

**Published:** 2019-10-03

**Authors:** Fiacre E. Rougieux, Wolfram Kwapil, Friedemann Heinz, Manjula Siriwardhana, Martin C. Schubert

**Affiliations:** 1 The University of New South Wales, School of Photovoltaic and Renewable Energy Engineering, Sydney, NSW2052 Australia; 2 Fraunhofer ISE, Heidenhofstr. 2, 79110 Freiburg, Germany; 3 The Australian National University, Research School of Electrical, Energy & Materials Engineering, Canberra, ACT2601 Australia

**Keywords:** Imaging techniques, Solar cells

## Abstract

In this paper we present a contactless transient carrier spectroscopy and imaging technique for traps in silicon. At each pixel, we fit the transient decay of the trap emission which allows us to obtain both the trap time constant and trap concentration. Here we show that this technique allows for high-resolution images. Furthermore, this technique also allows to discriminate between the presence of thermal donors or oxygen precipitates in as-grown wafers, without requiring a thermal donor killing step.

## Introduction

Recent solar cell efficiency records use Czochralski (Cz) grown silicon wafers. Ring-like defects often plague Cz wafers thereby reducing the cell efficiency. Such defects are oxygen precipitates or thermal donors, both of which respond differently to thermal processing. Going towards high-efficiency and high-yield, it is critical to be able to differentiate between oxygen precipitate-related ring defects and thermal donors-related defects. This allows to better predict how thermal treatments affect the wafers. Photoluminescence imaging is the technique of choice to image the effective lifetime of silicon wafers^1^. Deep Level Transient Spectroscopy (DLTS) is the workhorse defect transient spectroscopy technique^2^. However, there is no technique that allows for both transient spectroscopy and imaging of defects in silicon. Some groups have used DLTS mapping technique^3^ but not in imaging mode.

Several groups have performed trap imaging using lock-in thermography before^4–6^ and more recently using microwave photoconductivity measurements^7^. Quasi-Steady-State PhotoConductance (QSSPC) and Thermography-based carrier density imaging measures the sum of the electron and hole concentration (weighted by the respective conductivities for QSSPC. On the other hand, photoluminescence imaging measures the product of the electron and hole concentration. Hence thermography-based techniques allow for accurate imaging of trap concentrations. Earlier analyses focused on traps in steady state with the illumination source, that is, fast traps. In silicon solar cells it is also critical to measure slow traps (traps that are have a longer emission time constant than the decay time constant of a typical flash used to measure solar cells). Measuring slow traps is crucial as such traps can affect a range of solar cell characterization methods and can also impact on solar cell operation^8–11^. The thermography-based methods used in previous studies are unable to image slow traps as only the steady state signal is used to image traps whilst here we use the transient signal. Thus this work focuses on the imaging of slow traps in silicon wafers.

Here we show that Free Carrier Emission is a powerful tool to image slow traps and in particular thermal donor-related traps in Cz silicon wafers. We obtain trap concentration images and trap time constant images using advanced pixel-by-pixel analysis of the frequency dependent signal. The trap time constant is calibrated based on the lifetime measured by Free Carrier Emission which is itself calibrated on the lifetime as measured by PhotoConductance (QSSPC). Finally, we show that the effective lifetime is anti-correlated to the slow trap concentrations.

## Results

### Effective lifetime, fast traps and slow traps

**Figure 1:**
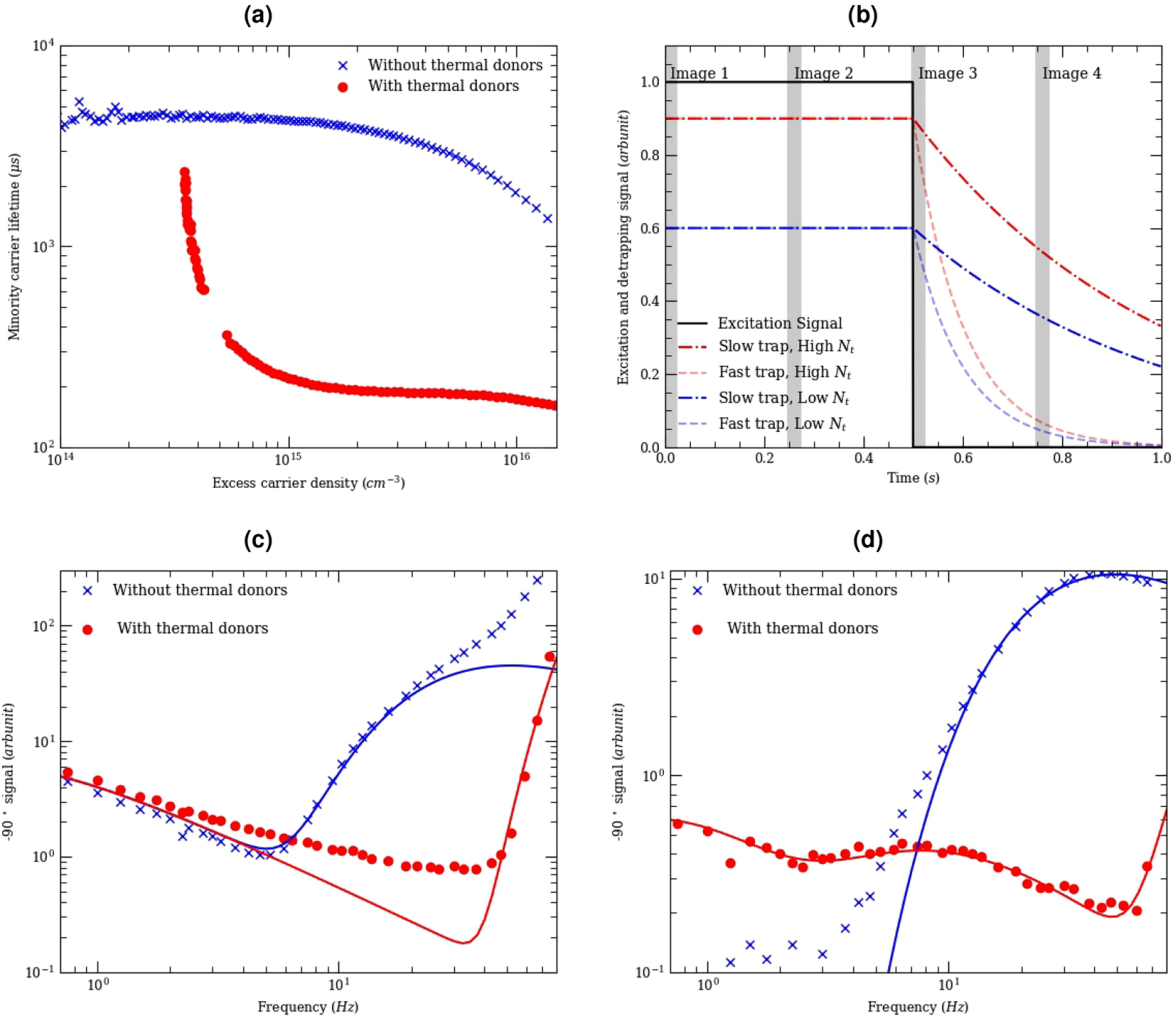
(**a**) Injection dependent lifetime as measured using Quasi-Steady-State PhotoConductance (QSSPC) showing steady-state trapping. (**b**) Principle of the lock-in system showing a range of detraping transients with different trap concentrations and time-constants during a single lock-in period. Frequency dependent lock-in free carrier emission signal measured under (**c**) high excitation power (9.1^−2^ *W*.*cm*^−2^) and (**d**) low excitation power (7.9 × 10^−6^ *W*.*cm*^−2^). The lines are fits with four parameters: a single value for the effective lifetime, the initial carrier density, a single value for the detraping time constant, the trap concentration.

In order to extract trap density images it is important to measure separately the effective lifetime. This allows to calibrate our images of trap densities. Figure 1a shows the minority carrier lifetime as a function of injection level for samples with and without thermal donors as measured by Quasi-Steady-State PhotoConductance (QSSPC) in transient and QSS mode^12^. The sample without thermal donor has a higher effective lifetime. At low-injection, the sample with thermal donor is affected by trapping effects. This is a well-documented effect, for instance see^13^. Note that the trapping effect observed in the photoconductance measurement is a fast trap effect as opposed to the slow trap effect describe in this paper.

### General principle of the technique

For a detailed description of the experimental setup, please see the Methods section of this paper. The imaging method adopted here is related to that of Ramspeck *et al*.^14^. Essentially, the method developed by these authors uses a dynamic measurement of the lifetime (as opposed to steady state method such as the QSSPC method above) to measure the lifetime and its spatial distribution. Dynamic measurements do not need external calibration of the lifetime as the results solely depend on measuring the transient kinetics of the carrier density signal. The work of Ramspeck *et al*. achieves this with lock-in free carrier absorption. For further information on this technique please see^14^. The work of Ramspeck *et al*. only focuses on measuring the minority carrier lifetime. However, here we determine both the effective lifetime and the trap density and emission time constant from dynamic lock-in thermography measurements. Note that here the measurement is performed at a temperature of 80 °C, so it is possible that the absolute value of the trap concetration might change but the trend will remain the same. Figure 1b represents the excitation signal (950 nm LEDs) and the measured signal (detrapping probed by free carrier emission). During one lock-in period, we measure four images at: 0 *s*; 1 × *t*_*period*_/4; 1 × *t*_*period*_/2 and 3 × *t*_*period*_/4. We apply a sin and -cos correlation function to retrieve the in-phase image (0° image) and out-of-phase image (−90° image). At four images per lock-in period, the sin and −cos function performs the simple function of adding or subtracting the images in this way:

1 $${0}^{\circ }image=image1+image2-image3-image4$$

2 $$-{90}^{\circ }image=-\,image1+image2+image3-image4$$

The 0° image (in-phase) represents the steady state carrier concentration and thus the effective lifetime. The −90° image encapsulates detrapping transients and effective lifetime transients. Figures 1c,d shows the −90° signal (averaged over an area of 3 × 3 *cm*^2^) as a function of lock-in frequency for samples with and without thermal donors. In such a graph, signals at high lock-in frequencies corresponds to kinetics with relatively small time constants (such as the lifetime) whilst signals at low lock-in frequencies corresponds to kinetics with relatively large time constants (slow traps). For instance, in our sample, the effective lifetime *τ*_*SRH*_ is 185 *μs* at Δ*n* = 2 × 10^15^ *cm*^*−*3^ and thus it impacts high-frequencies (1/(2 × *τ*_*SRH*_) = 2.5 kHz) with a tail all the way to 40 Hz. The detraping time is 40 ms (several order of magnitudes longer) and thus it impacts the low-frequency range (1/(2 × *τ*_*t*_) = 12.5 Hz). Within the lock-in period trap filling is virtually immediate (as evidenced by area averaged photoconductance measurements^15^), but detrapping is gradual. Assuming fast trap filling and that the lattice temperature is not changing between image1 and image2 (true if laser heating is weak), image 1 and image 2 are similar and thus Eq. 2 simplifies to:

3 $$-{90}^{\circ }image=image3-image4$$

The −90° image becomes the difference in the trapped carrier density at a fourth and three fourth of the lock-in period. This is comparable to a rate-window used in Deep Level Transient Spectroscopy measurements and in many respects the two evaluations are comparable. The −90° signal and its frequency dependence are therefore a direct measure of the trap concentration and trap time constant.

### Removing the impact of thermal transients

Figure 1c shows the −90° signal (averaged over an area of 3 × 3 *cm*^2^) as a function of lock-in frequency for samples with and without thermal donors. We use an excitation of 9.1^−2^ *W*.*cm*^−2^ here. At high lock-in frequency, the effective lifetime transients dominate the signal for both the sample with traps and without traps, hence we do not use the high frequency signal. The sample with traps has a lower effective lifetime as shown by Fig. 1a and as explained above, the impact of the effective lifetime transient occurs at a higher lock-in frequency for this sample. Note that at high lock-in frequencies the fit is poor for the sample without thermal donors. This is because of injection dependent effects not taken into account in this simple simulation. At low lock-in frequency, the −90° signal is comparable for the samples with and without traps. Our fit shows that this is because with an excitation of 9.1^−2^ *W*.*cm*^−2^, both samples heat up during excitation and cool down when the excitation is turned off leading to a thermal cooling transient. This signal dominates the low lock-in frequency range and does not allow to measure slow traps and the assumptions of Eq. 3 are not valid anymore.

Figure 1d displays the −90° signal (averaged over an area of 3 × 3 *cm*^−2^) as a function of lock-in frequency for samples with and without thermal donors. Here we used an excitation of 7.9 × 10^−6^ *W*.*cm*^−2^. Again, at high-frequency, the effective lifetime transients dominate the signal for both the sample with traps and without traps. The sample with traps has a lower effective lifetime (Fig. 1a) and the effective lifetime transient appears at a higher lock-in frequency for this sample. At low lock-in frequency, the −90° signal is two orders of magnitude higher for the samples with traps. The detrapping transients now dominate the signal and allow to measure the slow trap concentration and its time constant. Here, at least two different traps with corresponding trap time constants are present in the sample with traps.

### Lifetime image, trap density image and trap time constant image

**Figure 2:**
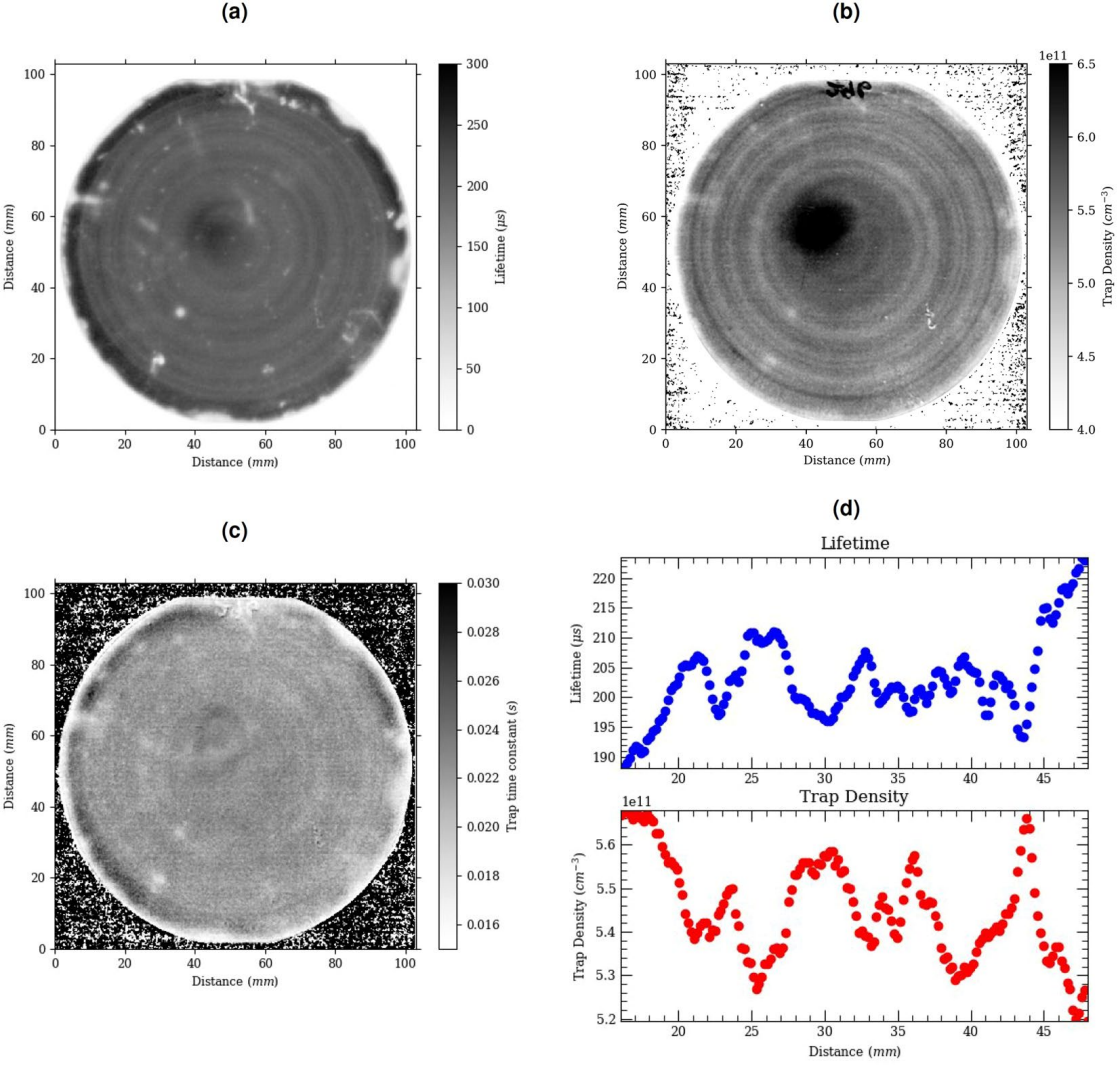
Sample with thermal donors. Image of the (**a**) effective lifetime (**b**) trap concentration (**c**) trap time constant. The sample without thermal donor is not shown here as the fit of the of the lock-in signal is just noise as shown in Fig. 1. (**d**) Profile of the effective lifetime and trap concentration. The dark area at the centre of the wavers in image a, b and c is related to the reflection of the cooled camera on the wafer. The camera is colder than the rest of the chamber which leads to an artifact that cannot be removed through the lock-in technique.

The 0° image calibrated using photoluminescence measurements allows to derive the effective lifetime image. Figure 2a shows the effective lifetime image displaying concentric rings characteristic of inhomogeneous oxygen distribution leading to inhomogeneous thermal donor concentration distribution.

We now determine the trap concentration and trap time constant image using the measured frequency dependent signal. We fit the frequency data at each pixel using a time constant and a scaling constant fed through a rate-window. Detraping occurs in the second half of the lock-in period in image 3 and 4. Because image 3 is measured at 1/2 the period and image 4 at 3/4 of the period, we fit the difference with this equation encapsulating the trapped carrier concentration at half and three quarter of the lock-in period:

4 $$-{90}^{\circ }signal=\alpha \ast [{N}_{t}\ast \exp (\frac{-1}{2\ast f\ast ta{u}_{t}})-{N}_{t}\ast \exp (\frac{-3}{4\ast f\ast ta{u}_{t}})]$$

Where *N*_*t*_ is the trap concentration, f is the lock-in frequency and *τ*_*t*_ is the trap time constant, there are four measurements per periods and thus the frequency is the frame per seconds/4. *α* is a constant determined by scaling the carrier density and minority carrier lifetime as measured from lock-in thermography at high frequency to the actual effective lifetime as measured by photoconductance. In doing so, we obtained a single value of *α* which can be used to quantitatively calibrate the trap concentration. Note that the lifetime from QSSPC was measured at 25 °C while the lock-in thermography measurement was performed at 80 °C. Temperature dependence of the lifetime may lead to a constant shift in our calibration. Ideally QSSPC measurement would be performed at the same temperature. Figure 1d shows a fitted experimental curve using the effective lifetime and two trap time constants. For the trap imaging below we fit the frequency curve between 5 Hz and 50 Hz to only measure the impact of a single trap. Figure 2b displays the resulting trap density image. This evaluation also allows us to derive the trap time constant at each pixel and generate a trap time constant image as displayed in Fig. 2c. Here the time constant is uniform throughout even when adapting the scale.

## Discussion

Figure 2d presents a linescan of the effective lifetime and the trap density along the radial direction of the wafer. The lateral variation in effective lifetimes and trap densities are anti-correlated. One could argue that the variation in the effective lifetime spatially leads to different trap filling concentrations or detrapping kinetics, in turn misleading us to observed a range of apparent trap concentration. This is not the case for two reasons. First if the effective lifetime is higher in a region, it should lead to higher excess carrier densities and in-principle more traps should be filled, thus effective lifetime and trap concentration should be correlated not anti-correlated. Second, the higher effective lifetime and corresponding higher carrier concentrations cannot affect directly the detrapping kinetics directly as we perform the measurement under low-injection conditions and the detrapping time constants are orders of magnitude higher than the effective lifetime. It is unclear if the trap is a direct participant in the recombination. The recombination channel and the trap could both be unrelated but a common consequence of varying concentrations of thermal donors.

In summary, traditional imaging methods focused on imaging fast traps in silicon. Here, we have developed a transient spectroscopy and imaging technique for slow traps in silicon. We analyze the frequency dependent signal (transient signal) from free-carrier detrapping with an analysis akin to rate-window based DLTS. This allows us to derive trap concentration images and trap time constant images.

## Methods

The samples were four inch n-type Czochralski-grown silicon wafers with and without thermal donors. We annealed sample 1 at 650 °C for 30 minutes to remove thermal donors and sample 2 at 450 °C for 96 hours to produce thermal donors. The sample without thermal donors had a resistivity of 0.84 Ω.*cm* and the sample with thermal donors had a resistivity of 0.25 Ω.*cm*. All wafers were later cleaned using RCA solutions, and Al2O3 layers were deposited using Plasma Atomic Layer Deposition for surface passivation. We use a mercury-cadmium telluride camera, with a frame rate of 300 frames per second. We operate at four images per period which caps our frequency to 75 Hz. We utilize an LED array with a wavelength of 950 nm as the excitation source. We lay the wafers on a mirror as illustrated here^16,17^. The wafers are at a temperature of 80 °C. Minority carrier lifetime measurements were performed using a Sinton WCT120 system^12^.
